# Slowbalisation in the Context of US-China Decoupling

**DOI:** 10.1007/s10272-022-1086-x

**Published:** 2022-12-16

**Authors:** Alicia Garcia-Herrero

**Affiliations:** Bruegel, Rue de la Charité/Liefdadigheidstraat 33, 1210 Saint-Josse-ten-Noode, Belgium

**Keywords:** F15, F32, F36, F41

## Abstract

While it is too early to confirm the depth and the sustainability of this new trend towards slower globalisation, it may be happening in more domains than we are fully aware of, at least for the near term given the renewed backdrop of the Russia-Ukraine war and the wider use of sanctions globally.

As probably the most prominent economic process in the 21st century, globalisation has attracted wide research interests and considerable support from academia for many years. But after decades of increasing globalisation in every aspect, from trade – pushed further by the growing role of value chains – to technology, movement of people and investment, it seems that the trend has turned towards slower globalisation (slowbalisation) which some have even called deglobalisation. Slowbalisation is not a new concept but rather a megatrend that has been seen before, for example, right before the First World War. We show evidence that slowbalisation, measured by decelerating trade and investment, as well as smaller global value chains, started in 2008 already. This trend seems to have accelerated due to the US push to contain China within the context of strategic competition between the two. Such containment is apparent not only in bilateral trade and investment flows but also in technology.

The COVID-19 pandemic has been a second very important factor pushing deglobalisation. The most obvious impact is in people-to-people movement, which, although recovering lately, still remains limited by preventative measures, particularly in mainland China, where a dynamic zero-COVID policy is in place.

Finally, the trend towards slowbalisation is much less evident for finance, with the exception of foreign direct investment, though increasing attempts to decouple particular types of financial flows from the US into China are emerging, including delisting Chinese companies from US stock exchanges and the imposition of sanctions for transactions with certain Chinese companies and individuals. All in all, it is too early to confirm the depth and the sustainability of this megatrend, but we should acknowledge that there are an increasing number of signals pointing in this direction.

**Alicia Garcia-Herrero**, Bruegel, Brussels, Belgium; and Hong Kong University of Science and Technology.

## Globalisation: The cornerstone of international economics turning the page

There are many benefits that economic research has attributed to globalisation, from higher economic growth to poverty reduction and even lower inflation. For instance, Khan and Riskin (2001) find that China's poverty reduction can be attributed to the opening up of its economy. Rogoff (2003) argues that the globalisation process helps push down inflation and any reversal of the free flow of production factors will re-introduce price pressure. In addition, Tomohara and Takii (2011) put forward that globalisation brings higher wages for local employers as foreign companies are given market access.

As a first step, it seems important to clarify what is meant by deglobalisation. Among the many definitions that can be found, we opt for a narrower view, related to economic factors, in particular a reduced number of exchanges, whether trade, investment, technology or movement of people. It should be noted that deglobalisation does not equate to economic decoupling, which refers to two specific economies reducing their economic linkages and, thus, their interdependence. Nevertheless, we consider if and how fast decoupling is happening between the US and China, given their increasing strategic competition (García-Herrero, 2018). We also consider how decoupling and deglobalisation interact.

Since 2008, the economic literature on globalisation has been less favourable. Hillebrand (2010), for example, argued that protectionism may improve income equality in some countries, although he still thought that a retreat from globalisation would lead to profoundly negative implications for the global economy. Even before the global financial crisis, the economic bedrock of globalisation, namely the link between trade and growth, was challenged. Rodriguez and Rodrik (1999) argued that the empirics of the trade and growth relationship are far from settled. Rodrik (2011) pushed the concept of the "globalisation paradox", by which globalisation will not be able to coexist with democracy and national self-determination. In other words, excessive government power would cause protectionism, while excessive market freedom would cause economic instability. The globalisation paradox seems to have become more visible lately based on the increasing number of trade disputes and government responses to severe shocks, including the COVID-19 pandemic. A few studies have attempted to measure the degree to which a de-

Figure 1
**Global trade value**
in %, year-on-year

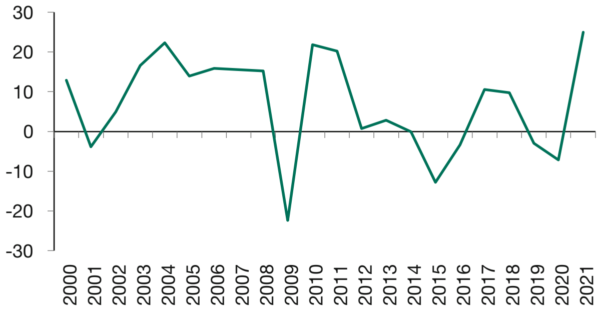

Sources: UNCTAD, Natixis.

Figure 2
**World global value chain participation**
in %

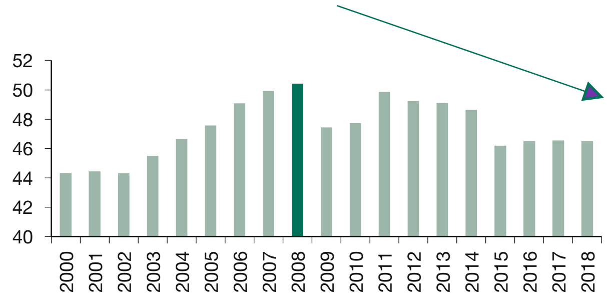

Note: Results for 2016-2018 are forecasted by UNCTAD-Eora.

Sources: UNCTAD-Eora database, Natixis.

Figure 3
**Global value chain participation in selected regions**

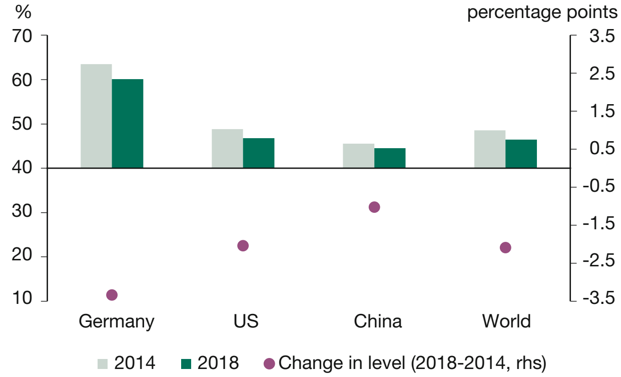

Note: Results for 2016-2018 are forecasted by UNCTAD-Eora.

Sources: UNCTAD-Eora database, Natixis.

globalisation process might be taking place, although most focus on trade (García-Herrero, 2018). Antràs (2020) found little systematic evidence to indicate that the world economy has entered an era of deglobalisation, but acknowledges that globalisation is continuing at a much slower pace.

To determine the phase of globalisation or deglobalisation we are currently experiencing, this contribution evaluates key aspects of exchanges, namely trade, global value chains, technology, movement of people and financial flows. The available data points to a slowdown in the globalisation process insofar as interlinkages are growing less rapidly. This is particularly the case for trade and investment. While it is still too early to assess whether the process is permanent, it seems important to measure the speed of the process for the different types of exchange (trade, technology, people and capital). Meanwhile, the sudden turn from engagement to strategic competition between the United States and China raises the question about the extent to which the two economies are decoupling, which feeds into the deglobalisation process we find in the data starting from 2008.

## Slowbalisation in trade well before the trade war

A slowing of global trade flows has been evident since the global financial crisis. This is noticeable in the trade in goods, in both value and volume, and also in the integration of global value chains. The movement of merchandise declined sharply during the 2008 global financial crisis, but the general expectation was that trade would thereafter continue to grow at rates similar to those prior to the crisis. But this has not been the case. Figure 1 shows that global trade value grew by an average of 2.7% from 2009 to 2018, a much lower rate than the 12.6% average growth before the global financial crisis.

The degree of integration of global value chains (GVC) has also declined since the global financial crisis. If this integration is measured by the value of intermediate goods that are either imported to be re-exported, or are exported to other countries for them to re-export, there has been a net decline since 2008 (Figure 2). The decline has been much more significant for Germany, Europe's exporting powerhouse, than for the US and China (Figure 3). The EU remains the world region most integrated into GVCs, but the decline in its participation is happening faster than for other regions and is in line with the EU's declining share of manufacturing exports at the global level.

Amid these changes, the World Trade Organization (WTO) has been weakened as the facilitator of global trade flows. Its appellate body, which arbitrates in disputes, has been functioning poorly, resulting partly from the greater heterogeneity of the WTO as more emerging countries have joined the club and the lengthy process to settle trade disputes.

Figure 4
**China's trade in goods with US**
in %, year-on-year

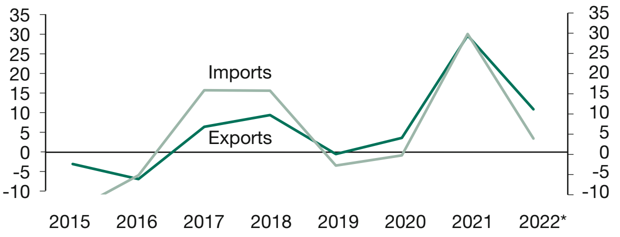

Note: * 2022 data as of October and compared to the first 10 months of 2021.

Source: General Administration of Customs, CEIC.

But more important has been the increasing confrontations in trade between the US and China. President Trump's profound disdain for multilateralism and China's state-led system are not compatible with the liberal nature of the global trading system and might have weakened the WTO's foundations. China has also been hit by US sanctions, which are being targeted against countries beyond Cuba, Iran and Russia. US sanctions against China are a further push towards their decoupling in trade, as well as in terms of technology and investment flows. US-China decoupling is reinforcing the post-global financial crisis deglobalisation trend, at least in terms of trade and global value chains.

The deglobalisation trend has clearly accelerated since 2019, ending in a collapse in trade flows at the peak of the COVID-19 pandemic (Figure 1). One of the reasons for the deceleration in trade before the pandemic was the US-China trade war and, consequently, the reduced trade flows between them, after a series of tit-for-tat protectionist measures (Figure 4). That said, there are some clear cyclical reversions towards more bilateral trade between the two since the COVID-19 outbreak, but the momentum is weakening again as the Federal Reserve aggressively hiked interest rates to tame inflation, which will likely reduce demand for Chinese goods.

In summary, the slowdown in globalisation trends is more notable for trade and global value chains, which have been shrinking and fragmenting since the global financial crisis.

## Technology protectionism still embryonic but more evident amid US-China decoupling

For years, the technology sector has been expanding globally with benefits in terms of economies of scale and network externalities. But such expansion could be deterred by policy constraints, as seen in the case of the technology decoupling between the US and China. In particular, the decoupling has sown the seeds of technology protectionism. In this section, we look at the various channels through which technology deglobalisation is happening, from export controls and screening of foreign investment, to bans on telecommunication software and hardware.

Firstly, transfer of technology has become increasingly restricted as global technology competition intensifies through export controls on high-end technology products. The US first developed the approval of the export of sensitive technology to tighten its control over technology transfer to the rest of the world. This was done through the reduction of export licenses for sensitive technological products. But as the geopolitical tension grew over the years, the approval rate of export licenses by the US Department of Commerce's Bureau of Industry and Security (BIS) for China declined from 82% in 2018 to 67% in 2021 (Figure 5). The value of goods exported to China subject to BIS authorisation fell 58% in 2021 versus 2018, with a larger share requiring a BIS license and can no longer be excepted (Figure 6). In turn, China has finally introduced

Figure 5
**US approval rate for export licenses**
in %

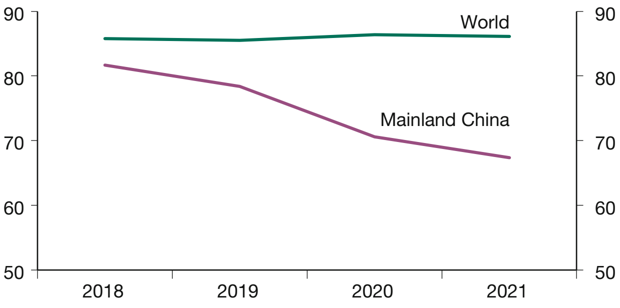

Sources: Natixis, US Department of Commerce's Bureau of Industry and Security (BIS).

Figure 6
**US export licenses for mainland China**

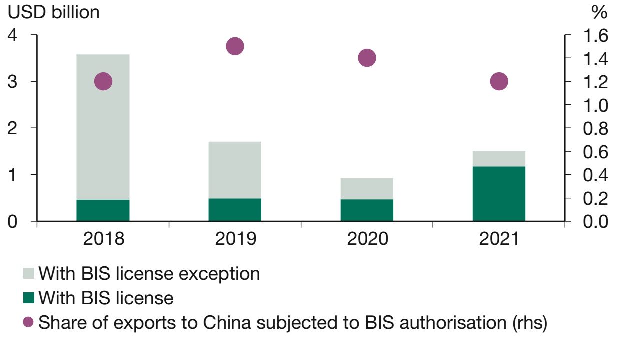

Sources: Natixis, US Department of Commerce's Bureau of Industry and Security.

export licenses for key technologies, including drones and artificial intelligence this year.

Beyond trade, the free flow of investment has also been limited, especially in relation to technology, because of increased investment screening. This is particularly the case for the US, after then President Donald Trump granted increased powers to the Committee on Foreign Investment in the United States (CFIUS) with the intent of blocking an increasing amount of Chinese mergers and acquisitions in the US, especially in the high-end industrial sector. The EU has followed and set up its own investment screening process in April 2020, pointing to technology protectionism globally, to counter China's move up the technology ladder. These moves show the unease in the west about China's increasing engagement in technological innovation. Western measures will only serve to drive technological decoupling.

More specifically for US-China competition, the US has introduced the so-called entity list,[1] which effectively forbids US companies from conducting business with the Chinese companies on the list. The US Bureau of Industry and Security published such a list of entities deemed risky to US national security as early as 1997, but the number of names on the list has expanded quickly since 2019, with the addition of Huawei and some of its affiliates and more Chinese corporations. In September 2020, China announced the release of its own identity list in retaliation, though the names of targeted companies had not been made public at the time of writing.[2] The grounds for listing targeted entities have been made public, including the taking of discriminatory measures against Chinese businesses on non-commercial grounds. Interestingly, the announced consequences of being on China's entity list are not sanctions, as is the case with the US identity list, but are rather being blocked entirely from trade and investment with China.

All in all, technology decoupling may eventually reinforce trade decoupling as the web of sanctions and prohibitions expands, and this is particularly the case for high value-added products with a large share of technology components. It goes without saying that trade decoupling between the world's two largest economies will foster deglobalisation of trade and, possibly, investment. One particular sector for which the impact of technology decoupling might be most serious is the semiconductor industry. This has become apparent with the US ban on sourcing semiconductors from Huawei, which affects not only American producers but also Taiwanese producers, among others. In September 2020, the US entity list, in addition to Huawei, added the largest producer of semiconductors in China (SMIC).

Another stumbling block in the US-China technology decoupling that has spilled over to the rest of the world is 5G technology. Since the US banned Huawei from providing 5G platforms in the US, other countries have followed, including the United Kingdom. The consequences of this move are still to be fully evaluated, but it looks like the world will end up with two different 5G ecosystems.

The US containment on Chinese technological expansion is also moving into software. In fact, the Trump Administration published an executive order targeting Chinese-owned social media platforms TikTok as well as WeChat in August 2020, but it was finally revoked by the Biden Administration in June 2021. The measures have threatened US residents and companies engaging in any transactions with these firms after the order is in effect. This is equivalent to the great firewall set up by China to block the free flow of information. But as the US follows China's lead, the internet and thus the exchange of global information are bound to bifurcate. In other words, the two previously mentioned ecosystems may be replicated in terms of hardware and standards in the software sector.

Beyond hardware and software, the next battle will clearly be the cloud and data storage. In fact, even with China's rapid boom in the digital market, it is important to note that it has never been fully integrated into the global economy (European Union Chamber of Commerce in China and Mercator Institute of China Studies, 2022). As a result, foreign companies operating in China have long faced immense market access restrictions imposed by the government, which can be generally divided into two dimensions.

The first is the decoupling of data management regimes arising from government regulations, which is affecting companies across all industries. As a consequence, cross-border data flows have been disrupted as different countries typically impose data localisation requirements for privacy and/or national security reasons. The best example is China's restrictions on data storage outside of China, which have been enforced since 2017, when China's Internet Security Law was first implemented. To address this, foreign data operators, such as Apple, now store Chinese user data in China through partnerships with local companies.

The second is the escalation in internet infrastructure scrutiny. This is especially relevant for companies caught between China's drive for greater technological self-reliance

1 See https://www.bis.doc.gov/index.php/policy-guidance/lists-of-parties-of-concern/entity-list.

2 See http://english.mofcom.gov.cn/article/policyrelease/questions/202009/20200903002580.shtml.

Figure 7
**Frequency of international passenger flights**
in % over 2019

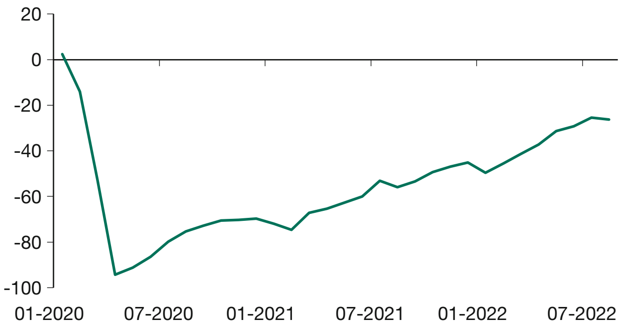

Sources: Natixis, IATA.

Figure 8
**COVID-19 stringency index**

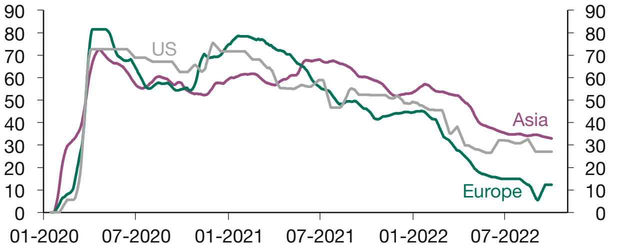

Note: Data as of 3 October 2022. 15 days moving average used. China (mainland), Indonesia, India, Japan, South Korea, Malaysia, the Philippines, Singapore, Thailand, Vietnam, Hong Kong and Taiwan are used as Asia proxies. Germany, the UK and the US are used as Europe proxies.

Sources: Natixis, OxCGRT.

and the US' aim to deter Chinese technology from a broad range of systems. Such regulation will affect all US businesses and push China to speed up the development of its own ecosystem in technology. In other words, upgrading the Chinese technology industry is more urgent than ever so China will not be deterred by the financial cost of supporting these industries.

## Mobility of persons slowed down

The number of international flights in 2020 plummeted because of global restrictions on mobility due to COVID-19 (Figure 7). While the number of international flight passengers has recovered to 50% of the pre-pandemic level (2019) since mid-2021, trends beyond the pandemic still show that international mobility has not returned to previous levels. Concerns about the impact of travel on health and the environment are likely to redefine the tourism industry. This is even more the case for business travel.

The good news is the suppression measures have begun to ease since mid-2021, led by Europe and the US, but not globally. For example, the COVID-19 stringency index shows that Asia has only caught up with the rest of the world in relaxing local and external restrictions since the beginning of 2022 (Figure 8). While the easier cross-border movement means a big rebound will come from international routes, which is a crucial difference between global and Asia-Pacific airlines, international tourists have only returned to 30% of 2019 on average in the Asia-Pacific, and the recovery will only become quicker (Figure 9).

## Financial slowbalisation is less pronounced but still noticeable

Increasingly, there are some early signs of financial deglobalisation. This has become more noticeable as the confrontation between the US and China has moved beyond trade with a growing number of conflicts in the financial sector. In this section, we examine globalisation trends through the lenses of foreign direct investment and portfolio investment.

The decline in cross-border capital flows is particularly evident in foreign direct investment (FDI), the most stable and possibly the most productive type of capital flow. Both inward (Figure 10) and outward FDI (Figure 11) flows as a share of global nominal GDP have been declining since the global financial crisis. This is especially true for outward FDI, which halved from 2.7% in 2008 to only 1.2% in 2018. This follows the trends of the decline in global trade and the fragmentation of global value chains, and could possibly be a consequence of those. Worse yet, the pandemic outbreak in 2020 witnessed a collapse in mergers and acquisitions arising from the cross-border constraints as well as the pause of many economic activities. While the weakening trend in 2020 reversed no-

Figure 9
**Asia's airports: International passengers**
in % over 2019

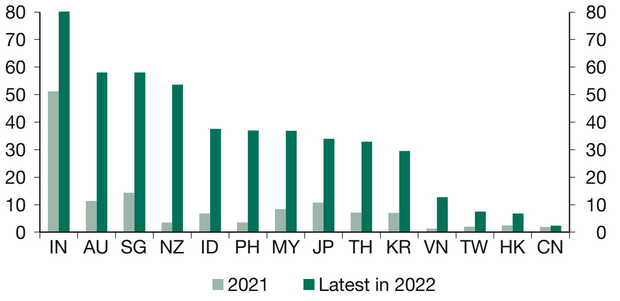

Note: Data as of July 2022 unless specified. Data as of June 2022 for Indonesia and the Phillipines.

Sources: Natixis, Various Airport Authorities and Tourism Boards, CEIC.

Figure 10
**World inward FDI flow**
in % of GDP

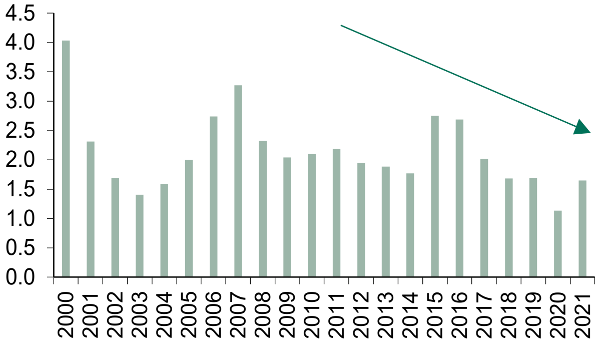

Source: UNCTAD.

Figure 11
**World outward FDI flow**
in % of GDP

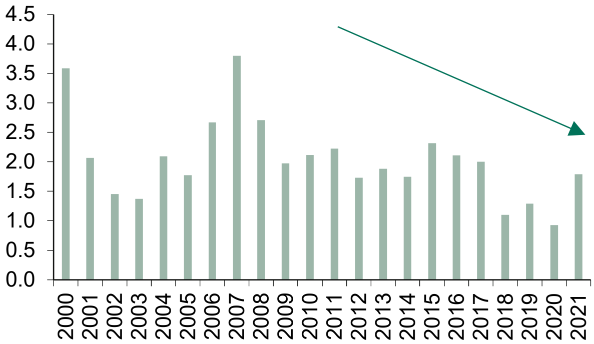

Source: UNCTAD.

ticeably in 2021, it is still much lower than the pre-global financial crisis level. It is hard to know whether FDI is no longer growing because of lack of demand, or because of constraints that make it harder for investors to operate. In any case, the difference in investment returns among recipient countries are such that the much reduced levels of FDI currently could be seen as a critical sign of the fragmentation of global capital markets.

As for portfolio flows, deceleration in bilateral portfolio flows has been more notable between the US and China, at least in terms of the holding of safe assets. In fact, China has been slowly but steadily downsizing its holding of US treasuries since its relationship deteriorated with the US during the trade war (Figure 12). As for the US, there is evidence of government attempts to decouple further. For example, the US State Department has asked universities to divest their holdings of specific Chinese assets, mainly related to Xinjiang or China's military-related companies.[3] As a result, there is a clear decrease in holding of Chinese long-term securities by US entities (Figure 13). The reduced ownership in Chinese assets is also seen by not just US investors, but more foreign investors because of the loss of confidence in Chinese assets due to regulatory changes (Figure 14).

In line with the reduction in cross-border lending, cross-border financing has become more difficult. For example, Chinese technology firms listed in the US have opted for secondary listings to avoid the risk of delisting from the US stock market. This has been done by Alibaba Group, JD.com, NetEase Inc. and more Chinese tech companies that have opted for secondary listings in Hong Kong. The Chinese government has meanwhile adopted policies to encourage the domestic funding of technology companies, including the launch in 2019 of the Science and Technology Innovation Board (SSE STAR Market) which has loosened regulations.[4] Based in Shanghai, the STAR Market has the objective of supporting promising technology start-ups in their equity financing, which helps to avoid US equity

3 https://www.state.gov/letter-from-under-secretary-keith-krach-to-the-governing-boards-of-american-universities/.

4 http://star.sse.com.cn/en/

Figure 12
**China's holding of US treasuries**
in USD trillion

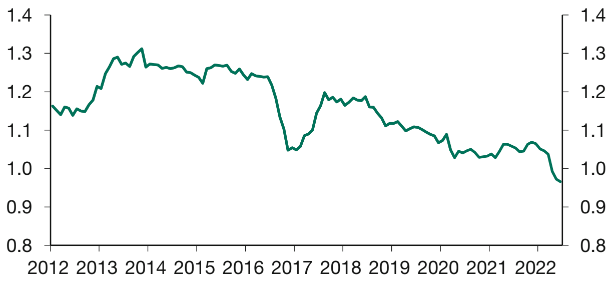

Source: TIC.

Figure 13
**US holding of Chinese long-term securities**
in USD billion

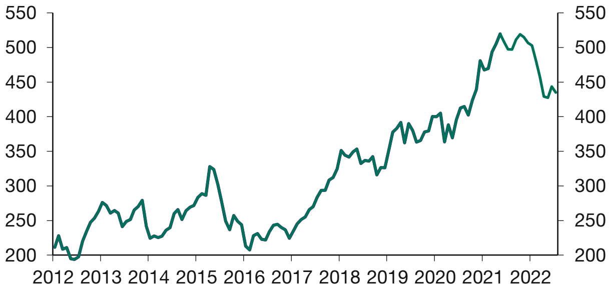

Source: TIC.

Figure 14
**Foreign ownership of Chinese bonds**

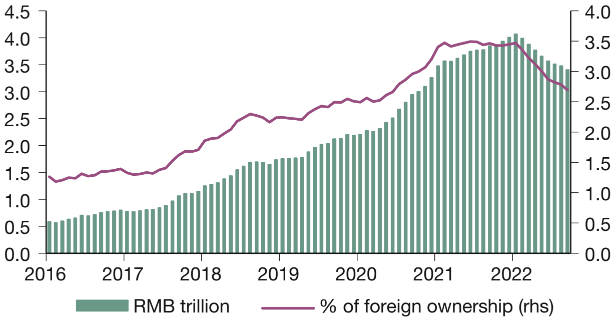

Note: Data as of July 2020.

Sources: China Central Depository & Clearing, Shanghai Clearing House, CEIC.

markets. China has also been increasingly selective in its choice of foreign banks in the arrangement of its sovereign issuance overseas. Since the renminbi has not yet become an international currency, China can use its sheer size in financial deals in screening market participants.

The slowbalisation trend is less pronounced than in other areas for financial flows, with the exception of FDI which is more closely linked to trade and the real economy. Nevertheless, the financial decoupling between the US and China is increasingly evident and is not only limited to FDI, though less FDI is significant. If the world returns to capital controls, there will be greater dislocation of global savings and, ultimately, lower potential growth.

## Conclusions

As probably the most prominent economic process in the 21st century, globalisation has attracted wide research interests and considerable support from academia for many decades. But after decades of increasing globalisation in every aspect, from trade – pushed further by the growing role of value chains – to technology, movement of people, and investment, it seems the trend has turned towards slowbalisation. The slowing of the globalisation process, after decades of growing globalisation since the reform of the international financial architecture after the Second World War, appears to have started in 2008, at least for trade, global value chains and foreign direct investment. The deceleration in trade and FDI globally has been fuelled recently by the strategic competition between the US and China; this competition is pushing them to decouple from one another, not only in terms of trade and FDI but, most notably, in technology. COVID-19 has been a second very important factor pushing slowbalisation. Beyond trade and FDI, movement of people has been an obvious victim of COVID-19.

The slowbalisation of trade is happening in terms of value and volume of gross trade and also in terms of the importance of global value chains. In other words, there are signs of a reduction in the exchange of intermediate goods between countries as a way to exploit comparative advantage and specialisation gains. Slowbalisation in trade and global value chains should not surprise us given the increasingly protectionist policies of a number of governments, notably the US, and others following the Russian invasion of Ukraine.

Beyond trade, technology decoupling between the US and China is seen in the reduction of approvals for export licenses, limits on use of hardware (through sanctions and the imposition of lists of companies with which the US and other companies cannot trade) and the attempts of outright bans on software. FDI flows are also shrinking, especially between the US and China. FDI screening is one obvious factor hampering FDI flows. International flows of people have seen sharper declines in the wake of COVID-19, which is recovering but far from completely given China's adoption of the dynamic zero-COVID strategy and the sheer size of its population. Finally, the trend towards deglobalisation is much less evident for finance, with the exception of FDI, though increasing attempts to decouple particular types of financial flows are emerging, including pressure to delist Chinese companies from US stock exchanges and the imposition of sanctions for transactions with certain Chinese companies and individuals. While it is too early to confirm the depth and the sustainability of this new trend towards slower globalisation, it may be happening in more domains than we are fully aware of, at least for the near term given the renewed backdrop of the Russia-Ukraine war and the wider use of sanctions globally.
